# Divalent Metal Uptake and the Role of ZIP8 in Host Defense Against Pathogens

**DOI:** 10.3389/fcell.2022.924820

**Published:** 2022-06-27

**Authors:** Derrick R. Samuelson, Sabah Haq, Daren L. Knoell

**Affiliations:** ^1^ Division of Pulmonary, Critical Care, and Sleep, Department of Internal Medicine, College of Medicine, University of Nebraska Medical Center, Omaha, NE, United States; ^2^ Department of Pharmacy Practice and Science, College of Pharmacy, University of Nebraska Medical Center, Omaha, NE, United States

**Keywords:** zinc, manganese, zinc transporter, host defense, infection. (Min. 5-Max. 8)

## Abstract

Manganese (Mn) and Zinc (Zn) are essential micronutrients whose concentration and location within cells are tightly regulated at the onset of infection. Two families of Zn transporters (ZIPs and ZnTs) are largely responsible for regulation of cytosolic Zn levels and to a certain extent, Mn levels, although much less is known regarding Mn. The capacity of pathogens to persevere also depends on access to micronutrients, yet a fundamental gap in knowledge remains regarding the importance of metal exchange at the host interface, often referred to as nutritional immunity. ZIP8, one of 14 ZIPs, is a pivotal importer of both Zn and Mn, yet much remains to be known. Dietary Zn deficiency is common and commonly occurring polymorphic variants of ZIP8 that decrease cellular metal uptake (Zn and Mn), are associated with increased susceptibility to infection. Strikingly, ZIP8 is the only Zn transporter that is highly induced following bacterial exposure in key immune cells involved with host defense against leading pathogens. We postulate that mobilization of Zn and Mn into key cells orchestrates the innate immune response through regulation of fundamental defense mechanisms that include phagocytosis, signal transduction, and production of soluble host defense factors including cytokines and chemokines. New evidence also suggests that host metal uptake may have long-term consequences by influencing the adaptive immune response. Given that activation of ZIP8 expression by pathogens has been shown to influence parenchymal, myeloid, and lymphoid cells, the impact applies to all mucosal surfaces and tissue compartments that are vulnerable to infection. We also predict that perturbations in metal homeostasis, either genetic- or dietary-induced, has the potential to impact bacterial communities in the host thereby adversely impacting microbiome composition. This review will focus on Zn and Mn transport *via* ZIP8, and how this vital metal transporter serves as a “go to” conductor of metal uptake that bolsters host defense against pathogens. We will also leverage past studies to underscore areas for future research to better understand the Zn-, Mn- and ZIP8-dependent host response to infection to foster new micronutrient-based intervention strategies to improve our ability to prevent or treat commonly occurring infectious disease.

## 1 Introduction

### 1.1 Zinc and Manganese in Host Defense Against Infection

#### 1.1.1 Zinc and Infection Prevalence

Within the vertebrate host, zinc (Zn) is the second most prevalent trace metal (Kehl-Fie and Skaar, 2010). Zn has a role in cellular metabolism and is a key component of proteins involved in cell structure and membrane stability (Maret and Li, 2009; Bonaventura et al., 2015). It enables immune defense, protein and DNA synthesis, wound healing, cell division and proliferation (Prasad, 1995; Maret and Li, 2009; Bonaventura et al., 2015). As a result, Zn nutritional deficiency has been associated with a variety of disorders, including immunological diseases. Studies in the 1960s first recognized the importance of Zn in host defense against infection. These assumptions were made due to records of premature death in Zn-deficient dwarfs, where infection was assumed to be the cause of mortality (Prasad et al., 1963).

Over the years, epidemiologic and clinical studies on the impact of Zn deficiency revealed that Zn-deficient individuals are more susceptible to infection. In developing countries, insufficient Zn intake in children less than 5 years of age has been associated with new-onset upper respiratory tract and gastrointestinal tract infections (Caulfield et al., 1998; Black et al., 2013). In fact, perinatal Zn deficiency decreases the acquistion of maternal antibodies, as well as impairing development of the immune system (Caulfield et al., 1998).

The importance of Zn in the development and maintenance of immune function is further supported by the significant reduction in the incidence of respiratory infections, diarrhea, and bacterial infections in infants, children, adults, and sickle cell disease patients following Zn supplementation (Sazawal et al., 1998; Prasad et al., 1999; Hemilä, 2011). This is supported by experimental infectious diarrhea models that demonstrate a beneficial role of Zn supplementation akin to what has been observed in infection-induced diarrhea in humans (Bolick et al., 2014; Sarwar et al., 2017; Wiegand et al., 2017). Additionally, Zn has been shown to maintain the integrity of epithelial barriers, decrease leukocyte infiltration and cytokine expression, and reduce *E. coli* and cholera toxin translocation into epithelial cells (Bolick et al., 2014; Sarwar et al., 2017; Wiegand et al., 2017).

#### 1.1.2 Manganese and Infection Prevalence

Manganese (Mn) is an essential trace element that maintains a variety of physiological functions including immune function and host defense and is also an important cofactor for many enzymes. At the onset of infection or inflammation the concentration of Mn in the circulation and tissues is altered. Mn levels are increased in pleural fluid and sputum in patients with thoracic empyemata, asthma, or bronchiectasis (Domej et al., 2000; Gray et al., 2010). In contrast, the concentration of Mn in *Chlamydophila pneumoniae* infected thoracic tissue was significantly lower compared to uninfected tissue (Nyström-Rosander et al., 2009). Similarly, the plasma levels of Mn in children with chronic hepatitis B virus infection was lower than uninfected control (Balamtekin et al., 2010). Mn supplementation in animal models of infection have been shown to bolster host defense against infection (Gajula et al., 2011; Zhang et al., 2020; Pan et al., 2018). In contrast, other studies have shown that Mn supplementation can increase pathogen virulence while reducing the innate and adaptive immune function including phagocytosis, superoxide dismutase (SOD) activity, Th1 and Th2 cell numbers, and complement C3 activity (Wu et al., 2021). In summary, Mn has an important role in immune function and defense against pathogens. However, in comparison to Zn, much less is known and the examples above illustrate that systemic and tissue Mn levels may increase or decrease in response to infection and reveal that more research is warranted to determine the functional roles of Mn in innate and adaptive immune-mediated host defense.

### 1.2 Dietary-Induced Zinc and Manganese Deficiency

Zn deficiency is typically caused by insufficient dietary intake and impacts approximately two billion people world-wide. It is responsible for 4% of the global burden of morbidity and mortality in children under the age of 5 years (Black et al., 2008; Mocchegiani et al., 2013). Severe Zn deficiency in humans, which is uncommon in the United States, adversely impacts growth and immune function and symptomatically manifests as dermatitis, diarrhea, delayed bone maturation, and altered neurologic function (Gibson, 2012; Mocchegiani et al., 2013). Moderate Zn deficiency, which is most prevalent in developing nations but also common in the United States, is commonly caused by a diet lacking protein-based foods that are high in Zn (Gibson, 2012; Sapkota and Knoell, 2018). Increased consumption of phytates, abundant in grains, legumes, nuts and tubers, is also a major contributor of Zn deficiency, as phytates are known to adsorb Zn in the gastrointestinal tract thereby preventing systemic absorption (Gibson, 2012; Sapkota and Knoell, 2018). Metabolic factors also increase the incidence of Zn deficiency. For example, infancy, adolescence, pregnancy and lactation, malabsorption syndromes, and excessive losses through urine, and intestinal secretions, as well as aging and alcoholism are all associated with an increased incidence of Zn deficiency (Gibson, 2005; Gibson, 2012; Mocchegiani et al., 2013). Likewise, acrodermatitis enteropathica and sickle cell disease are two hereditary illnesses that cause Zn deficiency (Gibson, 2005). Excessive Zn intake and corresponding toxicity is relatively rare, although doses above 1 g/day can cause acute gastrointestinal symptoms, fever and lethargy (Brown et al., 2001). Chronic intake of high doses of Zn can also lead to reduced absorption of copper and iron, impaired hematologic and immune functions and drive abnormalities in lipoprotein metabolism (Brown et al., 2001). Therefore, the upper limit of dietary intake has been set to 25–40 mg/day and the recommended dietary allowance (RDA) for Zn in adults is 11 mg/day for males and 8 mg/day for females (Mocchegiani et al., 2013).

The majority of Zn in humans (90%) is contained primarily in muscle, bone, liver, and other organs (Brown et al., 2001). Plasma contains less than 0.2% of the total body Zn content and adults have an average concentration of 15 μmol/L (corresponding to approximately 100 μg/dl) (Brown et al., 2001). Serum or plasma Zn levels have been used to assess the Zn status although it is often unreliable especially when acute or chronic inflammatory conditions are present (Brown et al., 2001; Mocchegiani et al., 2013). For example, increasing age, pregnancy, infection, hypoalbuminemia, surgery, and excessive exercise alter plasma Zn levels (Brown et al., 2001; Mocchegiani et al., 2013). In general, due to a lack of clinical awareness and accurate diagnostic markers, Zn deficiency is commonly overlooked. Overall, accurate assessment of Zn status in humans remains an obstacle with ongoing research to find reliable biomarkers (Mocchegiani et al., 2013).

In contrast to Zn, there exist far fewer reports of Mn deficiency in humans. Experimentally induced Mn deficiency *via* decreased dietary intake can cause dermatitis, clotting disorders, hair reddening, decreased fertility and birth abnormalities, aberrant glucose tolerance, altered metabolism, impaired growth and bone formation, and skeletal deformities (Finley and Davis, 1999; Aschner and Aschner, 2005). Since Mn deficiency is not well documented in humans, there is no RDA for Mn. Instead, an intake of 2–5 mg/day for adults has been set as the estimated safe and adequate daily dietary intake (Finley and Davis, 1999).

Mn toxicity has been reported in humans specifically in miners that inhale Mn laden dusts (Huang et al., 1989). Consequently, an upper limit of Mn intake has been established at 10 mg/day (Finley and Davis, 1999). Most cases of Mn toxicity that have been reported were associated with neurological symptoms and classified as Mn-induced Parkinsonism (Calne et al., 1994). Similar to Zn, there are currently no clinical standardized assays for the assessment of Mn status in humans. Experimentally, Mn concentrations in whole blood, magnetic resonance imaging of the globus pallidus of the brain, lymphocyte Mn superoxide dismutase (SOD) activity, and blood arginase activity have been studied (Greger, 1999). Overall, there are still no accurate, clinically accepted, standardized methods to assess Zn and Mn status in humans demanding further investigation in this area.

### 1.3 Zinc and Manganese in Immune Function

#### 1.3.1 Innate Immunity

New roles for Zn as a regulator of innate immune function continue to be identified in a variety of cells that include but are not limited to polymorphonuclear neutrophils (PMNs), monocytes, macrophages and dendritic cells (DCs) (Bonaventura et al., 2015; Maares and Haase, 2016; Sapkota and Knoell, 2018). Zn influences a broad range of cellular processes that include chemotaxis, phagocytosis, neutrophil extracellular traps regulated cell death (NETosis), apoptosis, and production of superoxides, cytokines and chemokines (Maares and Haase, 2016). Zn can also act as a chemoattractant and mediate phagocytosis *via* the Zn protein, early endosome antigen 1 (Hujanen et al., 1995; Simonsen et al., 1998).

Following phagocytosis, granulocytes and macrophages kill pathogens by the generation of nicotinamide adenine dinucleotide phosphate (NADPH) oxidase mediated reactive oxygen species (ROS) formation (Maares and Haase, 2016). Physiological concentrations of Zn are required for generation of ROS, whereas both lack of or excessive amounts of Zn inhibit NADPH oxidase (Hasegawa et al., 2000; Wessels et al., 2013). Analogous to Zn, studies have shown that Mn also exerts bactericidal activity by maintaining the optimal function of NADPH oxidase and myeloperoxidase-hydrogen peroxide-chloride antimicrobial system within neutrophils (Wu et al., 2021).

However, opposing data suggest that both Zn and Mn act as anti-oxidants that reduce the generation of ROS *via* the antioxidant functions of *via* both SOD and non-SOD-based mechanisms. SODs are an ubiquitous family of enzymes that catalyzes the dismutation of superoxide radicals into less harmful species, which are then removed by catalase and glutathione peroxidase (Zelko et al., 2002). There are three types of SOD; Cu/ZnSODs, found in the cytoplasmic space that uses copper or Zn for catalysis; nickel-containing SODs; and the Mn/FeSOD family that uses either Mn or iron and are found exclusively in the mitochondrial space (Zelko et al., 2002; Aguirre and Culotta, 2012). Previously, in a non-mammalian model, it was observed that Zn and Mn dependent SODs work in synergy to maintain the redox balance for immune defense against infection (Lu et al., 2015). However, whether the two types of SOD work in synergy in mammals is yet to be determined.

Zn deficiency promotes increased differentiation of monocytes into macrophages (Dubben et al., 2010). The polarization of macrophages into the pro-inflammatory M1 and anti-inflammatory M2 subtypes depends on Zn and phosphorylation of signal transducer and activator of transcription (STAT) 6. In a murine colitis model, inadequate Zn aggravated colonic inflammation by increasing the proportion of M1 macrophages in the colon with excessive Th17 cell activation (Higashimura et al., 2020). Knockdown of the Zn transporter, ZIP7, which leads to reduced Zn uptake in macrophages, drove macrophage differentiation toward an M2 phenotype and reduced production of interleukin (IL)-6 and tumor necrosis factor (TNF)-α (Xie et al., 2020). In another study, Zn treatment of THP-1 cells inhibited M2 polarization whereas differentiation into M1 macrophages was promoted by both physiological doses of Zn treatment and Zn deficiency (Dierichs et al., 2018). Thus, it is evident that Zn has a role in determining macrophage phenotype and function; however, results varied across studies likely as a consequence of the model and study conditions. Whether Mn is involved in macrophage differentiation remains to be determined.

Zn has both stimulatory and inhibitory effects on the secretion of cytokines from monocytes and macrophages depending on the dose, duration, and cell type. In human peripheral blood mononuclear cells (PBMCs), incubation with high Zn concentrations (100 mM) stimulated the release of pro-inflammatory cytokines, IL-6, IL-1β, and TNF-α (Wellinghausen et al., 1997; Maares and Haase, 2016). Zn also potentiated the effects of lipopolysaccharide (LPS). Specifically, when cells were incubated under “nonstimulatory concentrations” of Zn (<100 µM Zn) treatment with LPS lead to increased secretion of cytokine, IL-1β, compared to LPS treatment alone (Driessen et al., 1995). In contrast, low Zn levels inhibited monocyte activation caused by the superantigens, staphylococcal enterotoxins A and E, the *mycoplasma* arthritidis-derived superantigen, but not by toxic shock syndrome toxin-1 (Driessen et al., 1995). Zn reduced the release of LPS-induced TNF-α and IL-1β in primary human monocytes and the monocytic cell line Mono Mac1 by inhibiting enzyme activity of phosphodiesterase (PDE)-1, 3, and 4 and thus increasing cyclic guanosine-3′,5′- monophosphate (cGMP) (Von Bülow et al., 2005). Furthermore, Zn deficiency in HL-60 cells, a promyelocytic cell line, potentiated phorbol 12-myristate 13-acetate (PMA)-stimulated release of IL-1β, TNF-α, and IL-8 by decreasing the levels of the Zn finger protein, A20 (Bao et al., 2003; Prasad et al., 2011).

The presence of Zn transporters such as, Zrt/Irt-like protein (ZIP) 6 and 10 on the surface of DCs indicate that intracellular Zn concentrations play a vital role in DC maturation and function. For example, during infection, altered expression of Zn transporters decreases levels of intracellular free Zn in DCs, which contributes to the upregulation of major histocompatibility complex class II and co-stimulatory molecules required for antigen presentation to CD4^+^ T cells (Kitamura et al., 2006).

Zn also provides catalytic and/or structural functions in different proteins including enzymes and transcription factors involved in immune defense against pathogens. More importantly, in immune cells labile Zn and Zn finger proteins (ZFPs) regulate signal transduction pathways, including the nuclear factor-κB (NF-κB) signaling pathway, now one of the most extensively studied Zn-regulated cell signaling pathways (Haase and Rink, 2014; Gammoh and Rink, 2017). Following toll-like receptor (TLR)-4 activation by LPS, a rapid Zn-dependent signal is generated leading to the activation of the myeloid differentiation primary response gene 88 (MyD88) pathway and the downstream production of cytokines *via* NF-κB (Haase and Rink, 2014). In contrast, Zn has been shown to inhibit toll/IL-1R domain-containing adapter inducing interferon (IFN)-β-mediated activation of IFN regulatory factor 3 (IRF3) signals resulting in decreased IFN-β-production (Brieger et al., 2013). Labile Zn also regulates signal transduction in immune cells through inhibition of dephosphorylating enzymes including protein tyrosine phosphatases, cyclic nucleotide PDE, and dual specificity phosphatases (Haase and Rink, 2014; Gammoh and Rink, 2017). For example, high Zn concentrations inhibit PDE activity, leading to elevated cGMP levels and protein kinase A (PKA) activation resulting in impairment of NF-κB activation (Von Bülow et al., 2007). One of the well-known Zn finger transactivating factors, A20, is an inhibitor of the TNF receptor and TLR initiated NF-κB pathways and ultimately inhibitor of cytokine release from T cells and monocytes (Prasad et al., 2011). Additionally, Zn upregulates the expression of A20, which inhibits NF-κB and the subsequent production of cytokines (Prasad et al., 2004).

Our lab has demonstrated the importance of Zn in monocyte and macrophage signaling pathways in response to infection. Using a polymicrobial mouse sepsis model dietary-induced Zn deficiency was shown to enhance Janus kinase (JAK)-STAT3 and NF-κB signaling in the lung and liver resulting in increased bacterial burden, overactivation of the inflammatory and acute phase response, and higher mortality (Bao et al., 2010; Liu et al., 2014; Sapkota and Knoell, 2018). Similar studies have been conducted in mice and humans with supplementation of Zn; however, results have been inconsistent and inconclusive with some recording lower mortality rates while others observed no difference compared to treatment control groups. Mixed results from these studies may be due to differences in the timing and dose of Zn administered, since in animal models Zn was administered before the onset of sepsis, whereas in human studies, Zn was supplemented after the onset of sepsis. Clearly, more well-controlled, large, randomized studies will need to be conducted to determine whether Zn has a beneficial, neutral, or detrimental role (Alker and Haase, 2018).

In contrast to Zn, which predominantly decreases the activation of proinflammatory pathways, Mn has been shown to promote inflammation *via* activation of the NF-κB pathway. Mn treatment of microglia cell lines, the resident macrophage of the brain, potentiated the effects of LPS leading to increased expression of cytokines, IL-6 and TNF-α, nitric oxide, inducible nitric oxide synthase and heme-oxygenase- 1 (Chang and Liu, 1999; Filipov et al., 2005; Dodd and Filipov, 2011). Similarly, administration of Mn to mixed astrocyte-glial cultures augmented the expression of inflammatory cytokines and chemokines *via* the NF-κB pathway (Popichak et al., 2018). In human monocyte-derived macrophages Mn alone or in combination with LPS resulted in NF-κB activation and the production of IL-1β, IL-6, IL-8, IFN-γ, and TNF-α (Mokgobu et al., 2015). In a related study, Mn was shown to induce the translocation of NF-κB into the nucleus by decreasing mitochondrial membrane potential through the production of mitochondrial ROS. ROS accumulation resulted in phosphorylation of the NF-κB inhibitor, IκBα, and p65 translocation into the nucleus (Barhoumi et al., 2004).

Unlike Zn, there are no known Mn containing transcription factors (Nebert and Liu, 2019). Rather, Mn mechanistically regulates physiological processes as a cofactor for a broad array of enzymes that include hydrolases (e.g., arginase), ligases (e.g., glutamine synthase), lyases (e.g., phosphoenolpyruvate decarboxylase), and transferases (e.g., glycosyltransferase).

Among the glycosyltransferases, β-1,4-galactosyltransferase, a Golgi enzyme essential for the synthesis of the carbohydrate moiety of glycoproteins require Mn as a cofactor (Riley et al., 2017; Nebert and Liu, 2019). Many proteins require post-translational glycosylation and perturbations in the Mn homeostasis have been shown to contribute to many congenital and acquired diseases. In particular, Mn deficient mice exhibit skeletal abnormalities due to reduced synthesis of N-acetylgalactosamine containing chondroitin sulphate (Park et al., 2015). In type II congenital disorder of glycosylation, an inherited disease, impaired Mn transport (*via* a defective ZIP8 variant; discussed more in depth later) reduced blood and tissue Mn levels adversely impacting glycosylation of vital proteins resulting in skull deformation, severe seizures, psychomotor retardation, and deafness (Park et al., 2015; Riley et al., 2017). Mn-dependent, post-translational glycosylation is also important for proper immune function and host defense against microbes as highlighted in a recent study involving Crohn’s disease (CD) (Nakata et al., 2020). A large human genome-wide association study (GWAS) study revealed that a commonly occurring variant of the SLC39A8 gene was highly associated with CD patients. Based on this, a mouse Slc39a8 A393T allelic equivalent knock-in (KI) model was generated that recapitulated Mn deficiency in the colon and impaired glycosylation of key proteins causing gut barrier disruption and increased inflammation (Nakata et al., 2020). Taken together, Zn and Mn each play important roles in innate immune development, maintenance, and activation although they have distinctively different footprint with regard to the mechanisms by which they influence cellular function. Clearly, both are required for host defense, but it remains unclear to what extent their impact is integrated overall in response to invading pathogens since very few if any studies have yet examined the influence of both in cell culture or animal studies, let alone clinical trials.

#### 1.3.2 Adaptive Immunity

Zn plays an important role in the development, differentiation and function of T and B cells thereby impacting host defense, as well as autoimmune diseases. Zn is required for lymphopoiesis as exhibited by thymic atrophy and T cell lymphopenia in Zn deficient children and related animal models (King et al., 2005; Maares and Haase, 2016). Zn deficiency over time induces glucocorticoid production that enhances apoptosis of premature T cells (Fraker et al., 2000; Fraker and King, 2001; Haase and Rink, 2014). Additionally, thymulin levels, a thymus specific hormone that is crucial for T cell differentiation, is reduced by Zn deficiency and corrected by Zn supplementation (Prasad, 2007; Maares and Haase, 2016). Zn also helps maintain the balance between different types of T cells. Zn deficiency decreases the production of Th1 cytokines (IL-2, IFN-γ, and TNF-α), whereas Th2 cytokines (IL-4, IL-10) are unaffected all of which drive a Th2 predominant environment (Beck et al., 1997; Prasad, 2000). Suboptimal Zn levels also reduce the recruitment of naïve T cells and the activity of CD8^+^ cytotoxic T cells, which predisposes Zn deficient individuals to infection (Beck et al., 1997). Furthermore, Zn suppresses the development of Th17 cells by inhibiting IL-6-induced STAT3 activation (Kitabayashi et al., 2010). Whereas Zn can also promote IL-2-mediated T cell proliferation (Kaltenberg et al., 2010 #1) Regulatory T cells (Treg) that suppress the inflammatory response are central to immune homeostasis. Zn also regulates hyper-responsive immune reactions by increasing the Treg numbers through transforming growth factor (TGF)-β1 signaling, increased forkhead box P3 (Foxp3) expression, and inhibition of histone deacetylase Sirt-1 mediated Foxp3 degradation (Rosenkranz et al., 2016b; Maywald et al., 2017). Zn-mediated Treg polarization also helps to temper allergic responses in the setting of transplant organ rejection in both mouse and *in vitro* models (Campo et al., 2001; Rosenkranz et al., 2016b; Rosenkranz et al., 2017). Similarly, an experimental model for multiple sclerosis, revealed that Zn supplementation increased Treg numbers in the central nervous system with a corresponding reduction of Th17 cells that attenuated inflammation (Kitabayashi et al., 2010; Rosenkranz et al., 2016a).

Zn and Mn have also been linked to T cell receptor (TCR) signaling. Multiple signaling pathways in T cells are regulated by cytoplasmic free Zn that acts as second messengers. Zn stabilizes the TCR signaling complex by facilitating the binding of the src kinase, Lck to CD4, which initiates tyrosine phosphorylation of ZAP70 and CD3d (Kim et al., 2003). TCRs when stimulated with superantigens presented by DCs, trigger the influx of Zn, which decreases the TCR threshold to respond to suboptimal antigenic stimuli by decreasing the recruitment of SHP-1 to the TCR activation complex (Yu et al., 2011). In addition, stimulation of T cells increases the expression of the Zn transporter on the lysosomal membrane resulting in increased cytosolic Zn, inhibition of calcineurin phosphatase activity and increased IFN-γ synthesis (Aydemir et al., 2009). Mn ions are essential for CD28 phosphorylation in human peripheral T cells (Hutchcroft et al., 1996). Mn can also regulate TCR signaling *via* activation of calcineurin (Wu et al., 2021). In Jurkat T cells, Mn in the presence of PMA promoted the activation of AP-1, upregulation of c-Fos and c-Jun, and increased IL-2 production (Tanaka et al., 2012). Furthermore, in the setting of TCR cross-linking, Zn was involved in tyrosine phosphorylation of the TCR-associated membrane signal transduction molecules Lck, LAT, ZAP70, PLCγ1, and SLP76, all of which modulated mitogen-activated protein kinase (MAPK) signaling through the c-Jun N-terminal kinase (JNK)/cJun pathway. Interestingly, TCR signalling was not regulated by Cu/ZnSOD enzyme (Gill and Levine, 2013).

Like T cells, B cell development is dependent on Zn. Zn deficiency rapidly depletes precursor and immature B cells with relatively no change in the pro and mature B cell populations. These differences were later explained by low expression levels of bcl-2 in pro and mature B cells that prevented apoptosis (Fraker et al., 2000; Fraker and King, 2001). In contrast to acute Zn deficiency, chronic Zn deficient mice are able to maintain B cell lymphopoiesis for an extended time period possibly due to the adaptation *via* increased Zn absorption and reduced excretion (King et al., 2005). Furthermore, Miyai and colleagues investigated the mechanisms underlying Zn-mediated lymphopoiesis using *ZIP10* ^*−/−*^ mice, a Zn transporter that imports Zn across the plasma membrane. *ZIP10* ^*−/−*^ mice had decreased levels of intracellular Zn, increased caspase activity and apoptotic cell death in both pro and pre B cells. *ZIP10* ^*−/−*^ mice also had significant reductions in B cell populations and serum immunoglobulin (Ig) levels, demonstrating that Zn homeostasis is critical for early B cell development and survival (Miyai et al., 2014). In the same model of *ZIP10* ^*−/−*^, it has also been shown that Zn modulates B cell function by regulating B cell antigen receptor (BCR) signal transduction (Hojyo et al., 2014). Similarly, mice with hypomorphic mutations in ZIP7, a Zn transporter that is expressed on the Golgi membrane, exhibited profound B cell immunodeficiency. B cells from ZIP7 hypomorphic mice had significantly decreased cytoplasmic free Zn, increased phosphatase activity and reduced BCR signalling (Anzilotti et al., 2019). In addition, several ZFPs including leukemia/lymphoma-related factor (LRF), B lymphocyte-induced maturation protein 1 (Blimp-1) and ZNF521 have been shown to modulate maturation of B cell lineage and humoral immune response (Solomons, 2013).

Overall, far fewer studies have been conducted with Mn and B cell development and function so its potential role(s) is much less clear. Mn does have a pro-apoptotic effect on activated tonsilar B cells, Epstein Barr virus (EBV)-negative Burkitt’s lymphoma cell lines (BL-CL) and EBV-transformed B cell lines in a caspase-dependent manner (Schrantz et al., 1999). In contrast, Zn has been shown to prevent apoptosis in B and T cells by directly inhibiting caspases 3, 6, 7, 8, and 9, with caspase 3 having a dominant role (Stennicke and Salvesen, 1997; Maret et al., 1999; Huber and Hardy, 2012). Collectively, past studies demonstrate vital roles for Mn and Zn in response to pathogens yet strikingly, each has distinct and in some cases, opposing effects on basic vital functions. More recently it has been shown that both metals enter cells through the zinc transporter ZIP8. Accordingly, we will now focus on cellular uptake as a common feature shared by both metals in order to begin to better understand how mammals orchestrate host defense.

### 1.4 Metal Transport in Mammals

Studies over the past two decades that involve metal ion transporters have created an ever-expanding mosaic of the coordinated action of uptake and secretion systems that achieve proper metal homeostasis for all tissues and cells in mammals. Metal transit occurs at the plasma membrane, as well as cellular organelle membranes through coordination of low and high affinity transporters that often act in concert to maintain metal balance (Hediger, 1997; Eide, 1998; Radisky and Kaplan, 1999). Metal ion homeostasis overall is governed by two evolutionary consequences. 1) Redox reactions that are fundamental life processes coupled to transition metals that are essential for the function of most proteins involved in redox reactions. This includes eukaryrotic and prokaryotic cells that ferociously compete for metal ions; and 2) essential cellular biological processes that generate toxic reagents that, when present in abnormal amounts, cause damage or dysfunction to proteins and nucleic acids. Consequently, in response to pathogen invasion, metal ion transporters provide effective tools to competitively acquire metal ions, and at the same time regulate or buffer the changing environment in favor of the host to mitigate potential damage and cell death induced by pathogen invasion.

Different transporters can generally be grouped into those that are driven by the chemical energy of ATP and those that are driven by electrochemical gradients of protons and other ions. Some of the systems include coupled transporters, one of high affinity and low capacity and the other of low affinity and high capacity (Eide, 1997). Transition and trace metals are typically grouped into two categories that include redox-active ions such as Fe^2+^, Cu^2+^, Co^2+^ and to a lesser extent Mn^2+^; and non-redox-active ions such as Ca^2+^ and Zn^2+^. The redox-active ions normally function in enzymes that directly participate in redox reactions and the conversion of active oxygen-containing components. Although Zn is redox inert, it has a propensity to interact with transcription factors and other enzymes and proteins, including metallothioneins, involved in cellular metabolism either as a catalyst, inhibitor, or binding reservoir in part because the presence of redox-active species in these spaces can lead to the promotion of radicals that result in tissue damage.

In contrast, Mn has no known interaction with transcription factors but does have direct impact on proteins involved in glycosylation. All these processes require coordination of defined amounts of specific metal ions at the right place and at the right time whether it be for day-to-day maintenance or, for example, mounting a host response against invading pathogens. Overall, the field of metal ion transporters is now in its third decade of study. Although much has been revealed relative to their collective role in human health and disease, much remains to be elucidated. For example, trace metals often share the same transporter with similar or different affinity for transit into or out of cellular compartments. Under normal conditions where metals are sufficiently available and transporters sufficiently functioning, balance is maintained. However, what happens when equilibrium is off balance due to metal deficiency or transporter dysfunction or both? Further, does either predispose the host to greater risk under these circumstances when challenged by an invading pathogen?

In this review, we will focus our attention primarily on the Zn transporter, ZIP8, that has been shown to efficiently transport Zn and Mn (as well as Fe^2+^, Se^+2^, Co^+2^, and Cd^2+^) into the cytosol of a variety of cells involved in host defense. ZIP8, is unique, relative to other family members, in that it is highly induced following pathogen recognition and required by myeloid-lineage, lymphoid, and parenchymal cells to maintain proper host defense against pathogen invasion as discussed later (Liu et al., 2013; Pyle et al., 2017a; Pyle et al., 2017b). We will also briefly touch on ZIP14, the closest homologue to ZIP8, and also a known transporter of Zn and Mn but with different expression patterns across different tissues. In addition, recent human GWAS studies have revealed that a frequently occurring ZIP8 variant allele that leads to defective intracellular metal transport (rs13107325; Ala391Thr risk allele), is strongly associated with inflammation-based disorders (Pickrell et al., 2016; Costas, 2018) and bacterial infection (Ye et al., 2014). When considering the high incidence of dietary Zn deficiency due to inadequate dietary intake and the relatively high frequency of ZIP8 variant alleles across populations, the potential impact on host defense through alteration of the innate and adaptive immune systems in vulnerable individuals becomes highly relevant and deserving of additional investigation.

#### 1.4.1 The Role of Zinc Transporter Proteins

Zn and Mn are both required for the growth and sustenance of eukaryotic and prokaryotic cells thereby effectively creating a “tug of war” for nutrient acquisition between host and microbe. Microbes also possess a variety of metal transporters to maintain normal function, as well as potentially evade host defense mechanisms, although this will not be discussed further in this review, please see the review by Johnstone and Nolan (2015) for a description of bacterial metal transport systems. Mammalian transmembrane spanning metal ion transporters serve as the primary conduit of micronutrient biodistribution once pathogen invasion has occurred in an effort to eradicate the pathogen (Rowland and Niederweis, 2012; Stafford et al., 2013). Transporter-mediated increases in eukaryotic cytosol metal ion content lead to disruption of protein function in pathogens, (Kühn, 1999; Rowland and Niederweis, 2012), and result in nutrient deprivation of elements essential for bacterial growth and survival (Nairz et al., 2010). It has also been shown that transporters can concentrate metal content in niches within the cell where microbes congregate thereby toxifying the local environment ridding the host of pathogen. Likewise, granulocyte macrophage-colony stimulating factor (GM-CSF) was shown to induce Zn sequestration within macrophages thereby enhancing elimination of Histoplasma capsulatum (Subramanian Vignesh et al., 2013 #2).

In particular, 10 Zn export proteins (ZnT_1–10_) and fourteen Zn import, Zrt-Irt-like-Proteins (ZIP_1–14_) control Zn homeostasis in mammals (Lichten and Cousins, 2009). Collectively, these highly conserved ZnT and ZIP family transporters regulate Zn homeostasis under normal and stress-induced conditions in humans and all other mammals, including mice. The Zn transporter ZIP8 was first discovered following its induction in monocytes in response to *Mycobacteria* (Begum et al., 2002). Bacteria-mediated ZIP8 induction in human monocytes resulted in the production of a membrane-bound, glycosylated, 140 kDa protein, which chaperoned extracellular Zn into the cytosol (Pyle et al., 2017a). The gene that codes for ZIP8, SLC39A8, is evolutionarily highly conserved in all vertebrates. The SLC39A8 gene is ubiquitously expressed with SLC39A8 expression occurring in most cell types, including pluripotent embryonic stem cells; with ZIP8 mediating the uptake of multiple cations including Zn^2+^, Mn^2+^, as well as Fe^2+^, Se^2+^, Co^2+^, and the toxic metal Cd^2+^. The extent of constitutive versus inducible SLC39A8 expression varies across cell types and different stimuli. Early transfection studies with ZIP8 cDNA utilizing cell culture models revealed that ZIP8 expression had Km values for Mn^2+^ that were slightly higher than Zn^2+^ with both determined to be the best physiological substrate for ZIP8 compared to other divalent cations including Fe^2+^ and Co^2+^ (He et al., 2006; Wang et al., 2012). The ZIP8 protein is expressed in most if not all mammalian tissues (https://www.proteinatlas.org/ENSG00000138821-SLC39A8/tissue). Transporter and metal uptake studies revealed that the complex moves across the cell membrane as a Metal^2+^/(HCO^−^) electroneutral species that does not require ATP.

Although a crystal structure remains to be elucidated, ZIP8 is predicted to be an eight-transmembrane protein under physiological conditions that is secreted from the Golgi-ER as a heavily glycolsylated protein, which is then trafficked predominantly to the cell-surface membrane (Liu et al., 2008). In addition, ZIP8 has been shown to be located in the membrane of intracellular organelles that include, (He et al., 2006; Wang et al., 2012), the Golgi body, (Kelleher et al., 2012; Park et al., 2015), lysosome, (Aydemir et al., 2009), endoplasmic reticulum, (Choi et al., 2018), and mitochondrial membranes (Riley et al., 2017). In the case of polarized cells such as kidney and lung epithelia, ZIP8 has been shown to be predominantly expressed on the apical surface (Besecker et al., 2008).

Whether expressed on the cellular or organelle membrane, the net result is increased Zn concentrations in the cytosol. Presumably the same occurs for Mn although this has been much less studied. It bares recognition that of the 14 ZIP transporters, ZIP14 is most evolutionarily closely related to ZIP8. Both are within the LIV-1 subfamily (Taylor and Nicholson, 2003) that are distinguished by a signature sequence (HEXPHEXGD) in TM domain V that is not found in other ZIP transporters. Interestingly, both transporters possess a glutamic acid (E) at this key position, which likely confers the ability to bind/transport metal ions other than Zn (Taylor et al., 2007). Both proteins are similar in length (489 vs. 462 amino acids) and possess about 50% similarity in composition with each having at least three N-linked glycosylation sites in the N-terminal region. They possess very highly conserved TM domains IV and V which have been proposed to comprise part of an ion channel (Eng et al., 1998). Tissue distribution is remarkably different with Slc39a14 gene expression highest in liver > duodenum > kidney/brain > testis, (Girijashanker et al., 2008), whereas Slc39a8 expression is highest in kidney ≥ lung > testis (Wang et al., 2007). Using a *Xenopus* oocyte system to assess the metal-ion substrate profile of ZIP14, it was revealed that it is able to transport Zn^2+^, Mn^2+^, Fe^2+^, and Cd^2+^, but not Cu^2+^ similar to what was previously shown for ZIP8 (Pinilla-Tenas et al., 2011).

In the context of development, Slc39a8 is expressed in mouse gastrula, (Harrison et al., 1995), and visceral endoderm (Moore-Scott et al., 2007) at gestational day 7.5 and a potential factor in cell differentiation in embryonic stem (ES) cells and embryogenesis, as well as later in adult life (Zhu et al., 2007). In sharp contrast, SLC39A14 is not expressed in ES cells providing strong evolutionary evidence that SLC39A14 arose from a gene-duplication event from the earlier gene, SLC39A8 (Nebert and Liu, 2019). For the remainder of this review, we will focus primarily on ZIP8 in part, because more investigation involving Zn and Mn transport has been conducted in the context of immune function, as well as more extensive GWAS studies identifying clinically relevant polymorphic variant alleles that are associated with pathogenic traits in humans have been studied with respect to ZIP8.

#### 1.4.2 The Role of ZIP8 in Zinc and Manganese Transport and Immune Function

Manipulation of the Slc39a8 gene in mice including Slc39a8-overexpressing, Slc39a8 (neo/neo) knockdown, and cell type-specific conditional knockout (KO) mouse lines have revealed multiple vital roles for ZIP8 in mammals. For example, the Slc39a8 (−/−) global KO mouse is embryonic lethal. Slc39a8 (neo/neo) hypomorphs die prematurely and exhibit severe anemia, dysregulated hematopoiesis, hypoplastic spleen, dysorganogenesis, stunted growth, and hypomorphic limbs. More recently, GWAS have revealed human SLC39A8-defective variants that exhibit striking similarities including defects in multiple organs, tissues, and cell-types, as well as influence the development of virtually every organ system and relevant to this review, the immune system. For a more comprehensive and up-to-date review of these traits and comorbid conditions please refer to an excellent review by Nebert and Liu (2019). Relevant to this review article, GWAS studies have revealed both Zn-mediated and Mn-mediated pathogenic traits in humans as a consequence of defective intracellular transport. More recently, a myeloid-specific Zip8 KO mouse model exhibited significantly increased morbidity and mortality in a pneumococcus model that was mediated in part by a defective Zn transport (Hall et al., 2021). Relevant to the A391T variant allele found in humans, a mouse Zip8 A393T KI model exhibits increased vulnerability to chemically induced inflammatory bowel disease (IBD) as a consequence of defective Mn transport, (Sunuwar et al., 2020), which is consistent with previous GWAS studies conducted in patients with IBD, discussed more in depth below.

Upon bacterial challenge and depending on cell type, ZIP8 translocates to plasma, endosomal and/or lysosomal membranes and transports Zn into the cytosol (Aydemir et al., 2009; Besecker et al., 2008). Zn and ZIP8 are coupled to antiapoptotic, as well as anti- and pro-inflammatory mechanisms demonstrating their critical roles in maintaining a balanced host response. One of the first examples revealed that ZIP8-mediated Zn transport into primary human lung epithelia protects against apoptosis (Besecker et al., 2008). To first reveal that Zn and ZIP8 coordinate cytoprotection to inflammatory stress, mRNA transcripts for all Zn transporters including ZIPs and ZnTs were screened following stimulation with TNFα. Of all 24 transcripts examined, only SLC39A8 mRNA was markedly induced by TNFα (Besecker et al., 2008). Increased SLC39A8 expression resulted in elevated intracellular Zn content and cell survival in response to TNFα whereas, siRNA silencing of SLC39A8 prior to cytokine treatment resulted in significantly more apoptosis.

More recently, using a novel myeloid-specific, *Zip8* KO model, a vital role of ZIP8 in macrophage and dendritic cell (DC) function was revealed following pneumococcal infection in the lung (Hall et al., 2021). Administration of *S. pneumoniae* into the lung resulted in increased inflammation, increased tissue damage, and increased bacterial dissemination leading to increased morbidity and mortality in *Zip8-*KO mice compared to wild type (WT) counterparts. This was associated with increased numbers of myeloid cells, cytokine production, and cell death. *In vitro* analysis of macrophage and DC function revealed deficits in phagocytosis and increased cytokine production upon bacterial stimulation that was, in part, due to increased NF-κB signaling. Strikingly, alteration of myeloid cell function *via* lack of ZIP8 resulted in imbalance of Th17/Th2 responses, thereby also potentially impacting adaptive immune function, although more investigation is required to determine the impact on the memory response. These results for the first time reveal a vital ZIP8- and Zn-mediated axis that alters the lung myeloid cell landscape and the host response against pneumococcus. As touched on previously, other groups have revealed that related Zn transporters are essential mediators of immune function in DCs, (Kitamura et al., 2006) B-lymphocytes, (Anzilotti et al., 2019), and T-lymphocytes (Colomar-Carando et al., 2019) clearly establishing the importance of Zn homeostasis in defense against harmful pathogens. It remains unclear from these recent animal studies whether ZIP8-mediated deficits in Mn also contributed to altered immune function and worse outcomes although knowing that Mn likely influences major pathways involved in immune function including the NF-κB, cGAS-STING, and TCR signaling pathways (Wu et al., 2021).

We have previously shown that intracellular transport of Zn *via* ZIP8, dampens LPS-induced inflammation and pro-inflammatory cytokine production (IL-6, IL-1β, TNFα, IL-8) in monocytes and macrophages through blockade of IKK and NF-κB activity, (Liu et al., 2013) and also blocks LPS-induced IL-10 expression and release in macrophages (Pyle et al., 2017a). The NF-κB signaling pathway participates in many cellular responses to a broad array of stimuli including but not limited to cytokines, free radicals, and bacterial or viral infections. SLC39A8 gene expression is induced by the transcription factor, NFKB1; resulting in a rapid influx of Zn into monocytes and macrophages and coordination of NFKB1-mediated transcriptional activation of downstream host defense factors including cytokines and chemokines. Importantly, the new pool of cytosolic Zn brought into the cell by ZIP8 then negatively regulates pro-inflammatory responses by means of Zn-mediated downregulation of IκB kinase (IKK) activity (Liu et al., 2013). Moreover, Slc39a8 (neo/neo) fetal fibroblasts exhibited decreased Zn uptake and increased NF-κB activation. Related to Zn itself, mice fed a Zn deficient diet exhibit increased inflammation in response to polymicrobial sepsis (Knoell et al., 2009). Collectively, these findings identify a negative feedback loop involving ZIP8 that directly controls innate immune function through coordination of Zn homeostasis and NFKB1 gene transcription. In other studies, Zn supplementation in culture medium decreased nuclear localization and activity of C/EBPβ, a transcription factor that drives IL-10 expression (Pyle et al., 2017a). It was concluded that Zn regulates LPS-mediated immune activation of human macrophages in a ZIP8-dependent manner, as well as lowering IL-10 levels; these findings further indicate that Zn-mediated homeostasis in macrophages plays a pivotal role in host defense against pathogens.

Phytohemagglutinin (PHA), which causes potent mitogen-inducing activation and proliferation of lymphocytes, was used to stimulate T cells grown in culture that were isolated from human subjects who had received oral Zn supplementation (15 mg/day) and T cell activation was assessed (Aydemir et al., 2009). Compared to volunteers not receiving oral Zn, those on Zn supplementation showed higher expression of PHA-activated interferon-γ (IFNγ)—indicating that Zn potentiates T cell activation. Similarly, Zn treatment of PHA-activated T cell cultures resulted in increased IFNγ expression. When SLC39A8 mRNA was knocked down by siRNA, decreased ZIP8 levels resulted in less T cell activation; transiently transfected ZIP8 overexpression led to enhanced T cell activation. These findings indicate that, along with the role in human monocytes and macrophages, ZIP8 also participates in Zn-mediated T cell activation. Clearly, ZIP8-mediated transport of both Zn and Mn are critical to host defense with much more known regarding Zn compared to Mn. Further, there currently exists a paucity of studies centered upon ZIP8 in relation to the tandem role(s) of both metals during host defense against infection whether it be with a focus on innate or adaptive immune function. It also remains unclear whether both metals complement each other to synergize host defense or possibly, compete with each other for transport thereby negating some of their beneficial effects.

### 1.5 Zip8 Polymorphic Variants and Inflammation-Based Disease and Infection

As mentioned above, recent human GWAS studies have revealed that a frequently occurring ZIP8 variant allele that leads to defective intracellular metal transport (rs13107325; Ala391Thr risk allele), is strongly associated with inflammation-based disorders (Pickrell et al., 2016; Costas, 2018) and bacterial infection (Ye et al., 2014). In this section we will highlight the current literature regarding our understanding and potential role(s) of the various polymorphic variants of SLC39A8 in human disease Nebert and Liu (2019).

#### 1.5.1 Association of the Ala391Thr Risk Allele and Clinical Disease

Following work from our group demonstrating the protective role of SLC39A8 against inflammation and cytotoxicity in human lung cells (Besecker et al., 2008), several GWAS have reported correlations between a *SLC39A8* genetic variant and various clinical disorders.

To date there have been seven single nucleotide variants (SNVs) of *SLC39A8* identified (Table 1) (Nebert and Liu, 2019) SLC39A8 variants include; 1) c.112G > C (p.Gly38Arg), 2) c.1019T > A (p.Ile340Asn), 3) c.97G > A (p.Val33Met), 4) c.1004G > C (p.Ser335Thr), 5) c.610G > T (p.Gly204Cys), 6) c.338G > C (p.Cys113Ser) and 7) c.1172C > T (p.Ala391Thr). The p.Ala391Thr (rs13107325) is the most well documented SNV of SLC39A8 and has been associated with alterations/disease of the cardiovascular system (HDL-Cholesterol levels, BMI, hypotension, coronary artery disease, atherosclerotic plaques, NT-proBNP levels, acute coronary syndrome, and cardiovascular death), (Speliotes et al., 2010; Teslovich et al., 2010; Waterworth et al., 2010; International Consortium For Blood Pressure Genome-Wide Association Studies et al., 2011; Willer et al., 2013; Johansson et al., 2016; Pickrell et al., 2016; Esslinger et al., 2017; Haller et al., 2018), the respiratory system (response to albuterol, and allergy), (Pickrell et al., 2016; Mak et al., 2018), the liver (inflammation and fibrosis), (Parisinos et al., 2020), and the gastrointestinal system (CD and IBD), (Li et al., 2016; Collij et al., 2019; Nakata et al., 2020), as well as with neurological disorders (Parkinson disease, schizophrenia, and cerebrovascular disease) (Carrera et al., 2012; Schizophrenia Working Group of The Psychiatric Genomics Consortium, 2014; Pickrell et al., 2016; Costas, 2018; McCoy et al., 2019). Conversely, to date the other rare SLC39A8 variants (p.Gly38Arg, p.Ile340Asn, p.Val33Met, p.Ser335Thr, p.Gly204Cys, and p.Cys113Ser) have only been reported to be associated with dysmorphogenesis and Mn-deficient hypoglycosylation (Boycott et al., 2015; Park et al., 2015; Riley et al., 2017).

**TABLE 1:** Minor Allele Frequencies of SLC39A8.

Single Nucleotide Variant	Minor Allele Frequency	Reference
Ala.391.Thr	• 0.05 in American populations	Sunuwar et al. (2020)
	• 0.08 in Northern European populations	
	• 0.14–0.25 in Ashkamzi Jewish populations	
	• Monomorphic in African and South Asian populations	
Gly.38.Arg	• 0.0001255 in European populations	Park et al. (2015)
Ile.340.Asn	• Unknown frequency	Boycott et al. (2015), Park et al. (2015), Riley et al. (2017)
Val.33.met	• Identified using penetrant autosomal recessive models with a rare disease allele frequency of 0.0001	
Ser.335.Thr		
Gly.204.Cys		
Cys.113.Ser		

#### 1.5.2 Ala391Thr Risk Allele Mechanistic Insight

While many mechanistic steps remain to be elucidated, several important studies have begun to determine the mechanisms by which the A391T SLC39A8 variant affects health. Several mechanistic studies involved in a variety of disease states have begun to be elucidated using expression of the A391T variant in a variety of human cell lines. To examine the role of the A391T variant in cardiovascular disease, HEK293 (Human Embryonic Kidney cells) expressing either WT SLC39A8 or the A391T SLC39A8 variant were cultured with Cd (Zhang et al., 2016). Surprisingly, following co-culture with Cd the cells that overexpressed the defective variant were found to have higher intracellular Cd levels, increased Cd-induced toxicity, increased phosphorylation of mitogen-activated protein kinase-1 (MAPK1), and elevated NF-κB activation when compared to cells expressing the WT variant. These results were then duplicated in vascular endothelial cells (Zhang et al., 2016). These data suggested that altered Cd uptake in vascular endothelial cells could explain, in part, lower serum HDL-Cholesterol levels, coronary artery disease, and hypotension that is associated with the A391T variant.

Further, to investigate the role of the A391T SLC39A8 variant in celiac disease (CD), both intestinal enteroids and overexpressing SLC39A8 variant in HEK cells were employed (Melia et al., 2019). First, it was demonstrated that ZIP8 expression in intestinal enteroids was dependent on IFNγ levels, which is consistent with the observed relationship between NF-κB and ZIP8. However, over-expression of the ZIP8 A391T variant in HEK293A cells resulted in increased TNFα-induced NF-κB activation compared to WT ZIP8, which is consistent with past studies indicating that in cells expressing the A391T variant there is a marked loss of the ZIP8/Zn-mediated negative regulatory NF-κB response (cells lose the ability to stop NF-κB signaling) (Melia et al., 2019). These data suggest that in the intestinal epithelial compartment the alterations to the negative regulation of NF-κB due to the A391T variant may contribute to CD pathogenesis *via* augmented inflammation.

Finally, to assess the impact of the A391T SLC39A8 variant on the neuroinflammatory response the A391T variant was overexpressed in both Chinese hamster ovary (CHO) cells and primary pyramidal neurons (Tseng et al., 2021). Both CHO and primary neuronal cells expressing the A391T SLC39A8 variant exhibited reduced zinc transport into the cell. Additionally, electrophysiological recordings from perturbed neurons revealed a significant reduction in N-methyl-D-aspartate (NMDA)- and α-Amino-3-hydroxy-5-methyl-4-isoxazolepropionic acid (AMPA)-mediated spontaneous excitatory postsynaptic potentials and a reduction in GRIND2A and GRIA1/A2/A3 receptor surface expression in cells with knockdown/deletion of SLC39A8. However, the phenotypes were rescued by re-expression of WT SLC39A8 or application of the membrane-impermeable Zn chelator ZX1, but not by re-expression of the A391T SLC39A8 variant (Tseng et al., 2021). Likewise, loss of ZIP8 resulted in decreased blood-brain barrier integrity, increased IL-6/IL-1β protein expression, and increased NF-κB expression following TNFα stimulation (Tseng et al., 2021). These data suggest that the A391T SLC39A8 variant is associated with decreased Zn transport into the cell culminating in altered glutamate and immune function and may explain, in part, the association of A391T SLC39A8 variant and schizophrenia.

Recently a human A391T (A393T in mice) KI mouse was developed (Nakata et al., 2020; Sunuwar et al., 2020). This model has become a critical tool in advancing our mechanistic insight regarding the role of the SLC39A8 A391T variant. Similar to observations from human GWAS studies, Zip8 393T-KI mice exhibit reduced Mn-levels in the blood (Sunuwar et al., 2020). These mice also exhibited abnormal tissue Mn homeostasis, with decreased content in liver and kidney with a reciprocal increase in biliary Mn, providing *in vivo* evidence of hypomorphic Zip8 function. Upon challenge in a chemically induced colitis model, male Zip8 393T-KI mice exhibited enhanced disease susceptibility. Homozygous mice expressing the variant allele had reduced triantennary plasma N-glycan species similar to a population-based cohort that possessed a genotype-specific glycophenotype hypothesized to be linked to Mn-dependent glycosyltransferase activity (Sunuwar et al., 2020). At the same time another group also developed a human A391T KI mouse model *via* alternative genetic methods (Nakata et al., 2020). Similarly, the SLC39A8 A391T mice exhibited Mn deficiency in the colon, which was associated with impaired intestinal barrier function and epithelial glycocalyx disruption with a corresponding increased sensitivity to epithelial injury and pathological inflammation in the colon (Nakata et al., 2020).

Taken together these data strongly support the critical role of ZIP8 in regulating inflammation *via* NF-κB, and that the commonly occurring SLC39A8 A391T variant results in increased NF-κB activation, due at least in part to impaired Zn-mediated, negative regulatory feedback. However, whether deficits in both Zn and Mn transport *via* ZIP8 enhance risk of infection and pathogenesis, or if there are additive, and synergistic effects with Zn and Mn during disease and inflammation remains to be determined.

#### 1.5.3 Dysmorphogenic Manganese-Deficient Hypoglycosylation Risk Alleles and Mechanistic Insight

Clinically, individuals carrying the SLC39A8 Gly38Arg variant exhibit variably low levels of Mn and Zn in blood, and elevated Mn and Zn levels in urine, as well as an impairment of the Mn-dependent enzyme β-1,4-galactosyltransferase, a Golgi enzyme essential for biosynthesis of the carbohydrate portion of glycoproteins (Park et al., 2015). Importantly, high-dose Mn therapy was found to be effective in reversing impaired galactosylation, which suggests a critical role of SLC39A8 for Mn uptake and normal glycosylation (Park et al., 2015). In addition, to determine the function of SLC39A8 mutants associated with congenital disorder of glycosylation (CDG) and Leigh syndrome, cell transfection studies were performed. Specifically, HeLa cells were transfected with cDNA encoding on the following SLC39A8 variants: 1) Gly38Arg, 2) Gly38Arg + Ile340Asn, 3) Val33Met + Gly204Cys + Ser335Thr, and 4) Cys113Ser (Choi et al., 2018). HeLa cells expressing WT SLC39A8 exhibited increased Mn uptake, while all four SLC39A8 variants exhibited significantly impaired Mn uptake into the cells; however no differences in Zn, Fe, or Cu uptake were observed. In addition, all four SLC39A8 variants failed to localize to the cell surface and were retained within the endoplasmic reticulum. Cells expressing the SLC39A8 variants also exhibited decreased Mn levels in mitochondria and MnSOD activity, which was accompanied by enhanced oxidative stress (Choi et al., 2018). These data suggest that severe Mn deficiency seen in subjects with CDG and Leigh syndrome patients is at least partially explained by stunted mobilization of SLC39A8 to the cell surface and defective Mn uptake. Further studies will be required to better understand differences in Mn and Zn transport, or lack thereof, with allelic variants in the context of host defense.

#### 1.5.4 Mechanistic Insight Gained From Zip8 Deficient Mice

Unfortunately, as previously stated, Slc39a8 (−/−) global KO mouse is embryonic lethal, and Slc39a8 (neo/neo) hypomorphs die prematurely thereby limiting the utility of these models to development studies. As an alternative, the development of several cell type-specific conditional KO mouse lines has revealed multiple vital roles for ZIP8 in mammals. For example, hepatic ZIP8 deficiency was associated with Se dysregulation, liver inflammation and fibrosis, and neoplastic changes consistent with hepatocellular carcinoma (Liu et al., 2018). Additionally, in a study evaluating the role of Zn homeostasis, Zn transporters, and Zn dependent transcription factors during osteoarthritis (OA) pathogenesis several key findings were described (Kim et al., 2014). First, SLC39A8-mediated Zn influx results in the upregulation of several matrix-degrading enzymes (MMP3, MMP9, MMP12, MMP13, and ADAMTS5) in chondrocytes (Kim et al., 2014). Interestingly, ectopic expression of Slc39a8 in mouse cartilage tissue caused OA-related destruction of cartilage; however, in chondrocyte-specific Slc39a8 (−/−) KO mice, surgically induced OA-related degradation of cartilage was suppressed, which was accompanied by lower levels of Zn influx and the matrix-degrading enzymes. In addition, metal-regulatory transcription factor-1 (Mtf1) was discovered to be essential for regulating Zn-dependent ZIP8-mediated catabolism, and genetic downregulation of Mtf1 in mice decreased OA pathogenesis (Kim et al., 2014).

### 1.6 Zinc Deficiency, Zip8, and Zip8 Variants and the Gastrointestinal Microbiota

Zn is also an essential trace metal for bacteria of the intestinal flora, in fact approximately 20% of the dietary intake of Zn is utilized by the intestinal microbiota (Smith et al., 1972). However, very little is known about the role of Zn (even less about Mn) regarding the maintenance and/or regulation of the composition of the intestinal microbiota. Given the increasingly important role that the intestinal microbiota plays in host health and immune homeostasis more research is needed to understand the complex interaction between dietary trace metals, the host, and the intestinal microbiota.

Several studies have evaluated the effects of both Zn-deficient diets and Zn-fortified diets in commercial-food animals and found a significant association between Zn status and the composition of the microbiome. Specifically, Zn deficiency in broiler chickens (Gallus gallus) was associated with a significant reduction in abundance of Firmicutes and an increase in Proteobacteria phyla, which were further characterized (genus levels) as a significant increase in *Enterococcus*, Enterobacteriaceae, and Ruminococcaceae abundance, as well as a decrease in the prevalence of Peptostreptococcaceae and *Clostridiales* (Reed et al., 2015). Conversely, several studies have examined the effects of Zn-fortified diets on chick microbiome diversity (Reed et al., 2018). Specifically, the abundance of Ruminococcus in chicks fed a Zn-fortified wheat diet was found to be a key genus in discriminating between Zn deficiency and Zn repletion (Reed et al., 2018). Similarly, supplementation of chicks with Zn bacitracin increased gut microbiota diversity, with a significant reduction in *Lactobacillus* and Eubacterium genus and an increase in the abundance of Clostridiales and Faecalibacterium (Crisol-Martínez et al., 2017). Finally, Zn hydroxychloride supplementation of broiler chickens significantly decreased total bacteria and *Bacillus* abundance, whereas *Lactobacillus* abundance was increased in parallel with cecal lactic acid production and up-regulation of intestinal tight junction proteins (indicators of intestinal health) (Nguyen et al., 2021).

Similarly, dietary exposure to coated ZnO in piglets resulted in a significant improvement in intestinal morphology and immunity, including increased villi length, elevated immunoglobulin A (IgA) levels, increased gene expression of IGF-1, occludin, zonula occludens 1, IL-10 and TGF-β1, as well as reduced gut microbiota diversity. Changes in microbiota diversity were characterized by a decrease in the relative abundance of *Lactobacillus,* and *Clostridium* and *E. coli*.; however, *E. coli* abundance was increased at lower doses and decreased at higher concentrations of coated ZnO in diets (Shen et al., 2014; Wang et al., 2019). Similar studies further demonstrate that high dietary ZnO supplementation in weaned piglets reduced the abundance of *Lactobacillus* genus, and especially *Lactobacillus acidophilus*, *Lactobacillus mucosae*, and *Lactobacillus amylovorus*. In addition, high-dose dietary ZnO supplementation to piglets was shown to significantly modulate ileal bacterial diversity and relative abundance of *Lactobacillus*, *Escherichia*, as well as other minor species. Specifically, the majority of Enterobacteriaceae were characterized by a significant Zn-induced increase in relative abundance. Additionally, bacterial species with relative abundance of >1%, Zn exposure resulted in a significant increase in *Weissella cibaria*, *Weissella confusa*, *Leuconostoc citreum*, and *Streptococcus equinus*. In contrast, the most abundant species *Leuconostoc reuteri* decreased from 45% to 18% in response to Zn exposure (Vahjen et al., 2011). Lastly, a significant increase in intestinal microbiota richness and relative abundance of Lachnospiraceae, with a parallel decrease in Ruminococcus flavefaciens was observed in response to coated nano ZnO supplementation (Liu et al., 2021). Interestingly, the effect of Zn on the gut microbiota in weaned piglets seems to be specific for intestinal sites. For example, ZnO nanoparticle (ZnONP) supplementation significantly reduced bacterial abundance and diversity in ileum with increases in *Streptococcus* and decreases in *Lactobacillus* numbers. In turn, cecal and colonic microflora biodiversity and abundance were increased, with a specific elevation in *Lactobacillus* numbers and a decrease in Oscillospira and Prevotella abundance. ZnONP-induced modulation of gut microbiome was associated with increased expression of tight junction and antioxidant proteins, as well as reduced IL-1β, TNFα, and IFNγ mRNA expression due to inhibition of NF-κB signaling, altogether resulting in lower incidence of diarrhea (Xia et al., 2017). Zn has also been shown to modulate microbial metabolite production in pigs. Specifically, ZnO supplementation significantly increased volatile fatty acids, acetate, and butyrate in the ileum. However, the increase in short-chain fatty acids (SCFAs) was dose dependent as low ZnO increased concentrations, while high ZnO concentrations lead to a decreased concentration (Pieper et al., 2012).

Surprisingly there are a limited number of studies that have described the effects of Zn deficient diets or Zn supplementation on the intestinal microbiota of laboratory rodents. One of the first studies to investigate the role of Zn and the microbiota, found that dietary Zn deficiency significantly affects gut microbiota of pregnant mice. Specifically, low dietary Zn significantly decreased the abundance of Proteobacteria and Verrucomicrobia, whereas Actinobacteria, Bacteroidetes, and Firmicutes phyla were increased. Importantly, the intake of Zn uptake inhibitors also significantly altered the composition of the gut microbiota, although the patterns were quite different. Changes in the gut microflora composition were associated with reduced claudin3 protein levels in the gastrointestinal tract, and increased hepatic LPS levels, (Sauer and Grabrucker, 2019), suggesting that Zn is required as a factor not only for gut microbiota homeostasis but for gut epithelial barrier function as well. Similarly, a recent study, which utilized a global Znt7 KO mouse model, found that Zn transport dysfunction results in altered microbiota biodiversity in a sex-specific manner (Kable et al., 2020). Specifically, Znt7^+/−^ and Znt7^−/−^ genotypes were characterized by increased abundance of Allobaculum and unidentified members of the family Coriobacteriaceae in females, but not males. These changes were likewise associated with distinct patterns of mucin production (upregulated in male and down-regulated in female mice), which may explain the observed differential effects on the composition of the intestinal microbiota (Kable et al., 2020). Finally, our group has recently demonstrated that myeloid-specific Zip8KO mice exhibit marked differences in the cecal microbial communities when compared to WT mice (Samuelson et al., 2022). Specifically, we found that the loss of ZIP8 expression in myeloid lineage cells resulted in significant differences in beta-diversity and specific bacterial taxa. Precisely, we found that bacteria from the genuses *Desulfovibrio* and *Intestinimonas*, as well as the families *Clostridiales Family_XIII* and Lachnospiraceae, were enriched in WT mice compared to Zip8KO mice. Conversely, bacteria from the genuses *Muribaculum*, *Erysipelatoclostridium*, *Mucispirillum*, *Parasutterella*, and Prevotellaceae*_UCG-001*, as well as from the family Ruminococcaceae were enriched in Zip8KO mice compared to WT mice (Samuelson et al., 2022). Most strikingly, upon a *S. pneumoniae* lung infection, mice recolonized with Zip8KO-derived microbiota exhibited an increase in weight loss, bacterial dissemination, and lung inflammation compared to mice recolonized with WT microbiota (Samuelson et al., 2022), which suggest that impaired Zn uptake not only influences the gastrointestinal microbiota, but that these changes also significantly influence host immune regulation. While most of the literature support the hypothesis that Zn deficiency or impaired Zn uptake alters the microbiome, one study did not observe any substantial alterations to the gut microbiota in mice with dietary Zn deficiency (Mayneris-Perxachs et al., 2016). Please refer to Table 2 for a review of the literature as it relates to effects of Zn and Zn-transporters on intestinal microbiota composition in mammals and birds.

**TABLE 2:** Effects of ZN and Zn-transporters on the intestinal microbiota composition.

Zn status	Bacterial taxa (phylum, class, family, genus, species) all taxa are classified to the lowest taxanomic level	Abundance change (relative to control)	Host species
Zn deficient	Firmicutes	Decreased	Broiler Chickens
	Proteobacteria	Increased	
	Proteobacterial, Gammaproteobacteria, Enterobacterals, Enterobacteriaceae	Increased	
	Bacillota, Clostridia, Clostridiales	Decreased	
	Bacillota, Clostridia, Clostridiales, Ruminococcaceae	Increased	
	Bacillota, Clostridia, Clostridiales, Peptostreptococcaceae	Decreased	
	Bacillota, Bacilli, Lactobacillales, Enterococcaceae, Enterococcus	Increased	
	Proteobacteria, Gammaproteobacteria, Enterobacterales, Enterobacteriaceae, Escherichia, coli	Decreased	Human (children)
	Bacillota, Clostridia	Decreased	
	Bacillota, Clostridia, Clostridiales, Ruminococcaceae, Subdoligranulum	Decreased	
	Bcillota, Negativicutes, Vellionellales, Veillonellaceae, Veillonella	Decreased	
	Bacillota, Negativicutes, Selenomonadales, Veillonellaceae, Megasphere	Decreased	
	Bacillota, Bacilli, Lactobacillales, Streptococcaceae, Stretococcus	Decreased	
	Bacillota, Bacilli, Lactobacillales, Lactobacillaceae, Leuconostoc	Decreased	
	Bacteroidota, Bacteroidia, Bacteroidales, Bacteroidaceae, Bacteroides	Decreased	
	Proteobacteria	Decreased	Mice (pregnant)
	Verrucomicrobiota	Decreased	
	Firmicutes	Decreased	
	Bacteroidetes	Decreased	
	Actinobacteria	Decreased	
Zn Fortifed (ZnONP)	Proteobacteria, Gammaproteobacteria, Enterobacterales, Enterobacteriaceae	Increased	Pigs
	Proteobacteria, Gammaproteobacteria, Enterobacterales, Enterobacteriaceae, Escherichia, coli	Decreased(high dose)	
	Bacillota, Bacilli, Lactobacillales, Lactobacillaceae, Lactobacillus	Decreased	
	Bacillota, Bacilli, Lactobacillales, Lactobacillaceae, Lactobacillus, acidophilus	Decreased	
	Bacillota, Bacilli, Lactobacillales, Lactobacillaceae, Lactobacillus, mucosae	Decreased	
	Bacillota, Bacilli, Lactobacillales, Lactobacillaceae, Lactobacillus, amylovorus	Decreased	
	Bacillota, Bacilli, Lactobacillales, Lactobacillaceae, Lactobacillus, reuteri	Decreased	
	Bacillota, Bacilli, Lactobacillales, Lactobacillaceae, Weissella, cibaria	Increased	
	Bacillota, Bacilli, Lactobacillales, Lactobacillaceae, Weissella, confusa	Increased	
	Bacillota, Bacilli, Lactobacillales, Lactobacillaceae, Leuconostoc, citreum	Increased	
	Bacillota, Bacilli, Lactobacillales, Streptococcaceae, Streptococcus, equinus	Increased	
	Bacillota, Clostridia, Clostridiales, Clostridiaceae, Clostridium	Decreased	
	Bacillota, Clostridia, Clostridiales, Clostridiaceae, Ruminococcus, flavefaciens	Decreased	
	Bacillota, Clostridia, Clostridiales, Lachnospiraceae	Increased	
	Firmicutes	Decreased	Human *in vitro* fermentation cultures
	Bacteroidetes	Increased	
Zn Fortifed (Zn Bacitracin)	Bacillota, Clostridia, Clostridiales	Increased	Broiler chickens
	Bacillota, Bacilli, Lactobacillales, Lactobacillaceae, Lactobacillus	Decreased	
	Bacillota, Clostridia, Clostridiales, Eubacteriaceae, Eubacterium	Decreased	
	Bacillota, Clostridia, Clostridiales, Clostridiaceae, Faecalibacterium	Increased	
Zn-Fortifed (Zn hydroxychloride)	Bacillota, Bacilli, Lactobacillales, Lactobacillaceae, Lactobacillus	Increased	Broiler chickens
	Bacillota, Bacilli, , Bacillales, Bacillaceae, Bacillus	Decreased	
Zip8 KO	Proteobacteria, Betaproteobacteria, Burkholderiales, Sutterellaceae, Parasutterella	Increased	Mice
	Thermodesulfobacteriota, Desulfovibrionia, Desulfovibrionales, Desulfovibrionaceae, Desulfovibrio	Decreased	
	Bacillota, Clostridia, Clostridiales, Clostridiaceae, Intestinimonas	Decreased	
	Bacillota, Clostridia, Clostridiales Family_XIII	Decreased	
	Bacillota, Clostridia, Clostridiales, Lachnospiraceae	Decreased	
	Bacillota, Clostridia, Clostridiales, Ruminococcaceae	Increased	
	Bacteroidetes, Bacteroidia, Bacteroidales, Muribaculaceae, Muribaculum	Increased	
	Bacteroidetes, Bacteroidia, Bacteroidales, Prevotellaceae_UCG-001	Increased	
	Firmicutes, Erysipelotrichia, Erysipelotrichales, Erysipelotrichaceae, Erysipelatoclostridium	Increased	
	Deferribacterota, Deferribacteres, Deferribacterales, Deferribacteraceae, Mucispirillum	Increased	
SLC39A8 A391T	Bacillota, Clostridia, Clostridiales, Lachnospiraceae, Anaerostipes	Decreased	Humans
	Bacillota, Clostridia, Clostridiales, Lachnospiraceae, Coprococcus	Decreased	
	Bacillota, Clostridia, Clostridiales, Lachnospiraceae, Roseburia	Decreased	
	Bacillota, Clostridia, Clostridiales, Lachnospiraceae, Lachnospira	Decreased	
	Bacillota, Clostridia, Clostridiales, Lachnospiraceae, Dorea	Decreased	
	Bacillota, Clostridia, Clostridiales, Clostridiaceae, SMB53	Decreased	
	Bacillota, Clostridia, Clostridiales, Ruminococcaceae	Decreased	

Finally, a limited number of studies have demonstrated the potential association between Zn status and the composition of the human gut microbiota. Specifically, *in vitro* reactors of the human colon microbiota demonstrated that ZnONP exposure at high concentrations significantly reduced the abundance of gut microbiota, as well as decreased bacterial biodiversity, SCFA production, and antibiotic resistance genes, which was associated with an increase in relative abundance of Bacteroidetes and a lower percentage of Firmicutes (Zhang et al., 2021). A preliminary study in a cohort of Pakistani children demonstrated that formula-fed children with Zn deficiency are characterized by lower abundance of *Escherichia*, as well as decreased relative number of Veillonella, *Streptococcus*, *Bacteroides*, Leuconostoc, Subdoligranulum, Megaspheare, and Clostridia (Durrani et al., 2021). However, correlation analysis did not reveal a strong association between serum Zn levels and intestinal bacteria (Durrani et al., 2021). Similarly, humans with the SLC39A8 A391T variant exhibited significantly altered gut microbiota communities, including reduced abundance of Anaerostipes, Coprococcus, Roseburia, Lachnospira, SMB53, Ruminococcaceae, Eubacterium, Dorea, and *Bacteroides*. The patterns of gut microbiota observed in SLC39A8 A391T variant carriers shared several similarities with those shown in patients with CD and obesity (Li et al., 2016). At the same time, another study did not reveal any significant association between SLC39A8 missense variant and gut microbiota, although SLC39A8 A391T risk allele was significantly associated with CD (Collij et al., 2019).

Taken altogether, these data demonstrate that Zn status has a significant impact on gut bacteria biodiversity, in food-production animals, rodents and human subjects (Table 2). Effects of physiological and nutritional Zn doses also result in improved gut wall integrity, thus contributing to reduced translocation of bacteria and gut microbiome metabolites into the systemic circulation. However, more research is clearly needed to not only understand the effects of Zn on the gut microbiota, but to gain a complete understanding of the downstream consequences of Zn-mediated intestinal microbiota changes on host defense against harmful, commonly occurring pathogens.

### 1.7 Summary

Mn and Zn to some extent, share chemical similarity, so it is not surprising that they also compete for uptake into eukaryotic cells *via* identical transport pathways. The same holds true for divalent transition and trace metal uptake in pathogens.

Environmental factors, primarily dietary intake, influence the abundance of Mn and Zn in humans. Manganese deficiency due to insufficient dietary intake is relatively unheard of whereas Zn deficiency is common through the world, often a result of low abundance in common food sources.

More recently, alteration of the gene that codes for the Zn transporter ZIP8, has shed new light on disease pathogenesis due to deficits in Mn and Zn intracellular uptake. Clearly, patients that harbor defective ZIP8 alleles that alter metal transport have been shown to have increased incidence of inflammatory-based diseases. One of the most studied is the defective A391T hypomorphic allele and IBD which results in defective glycoslyation and subsequent insufficient intracellular Mn uptake. Similar studies in humans that possess the variant allele and explore the potential impact of insufficient intracellular Zn uptake remain to be conducted but are warranted. Clearly, Mn and Zn are essential metals that humans require to carry out normal day-to-day functions as well as prepare the host to conduct battle against harmful pathogens. Despite the essentiality of both metals very few if any studies have determined their dual impact in host defense creating a number of important questions that remain to be addressed. For example, upon infection ZIP8 is rapidly induced and translocates to the plasma and organelle membranes to raise intracellular metal content. In the setting of Mn and Zn sufficiency and infection, does ZIP8 coordinate the spatial distribution of both metals simultaneously to enhance the impact of each in an additive or synergistic manner? Or, do Mn and Zn compete for uptake and by doing so, potentially mitigate the impact of one over the other?

**FIGURE 1:**
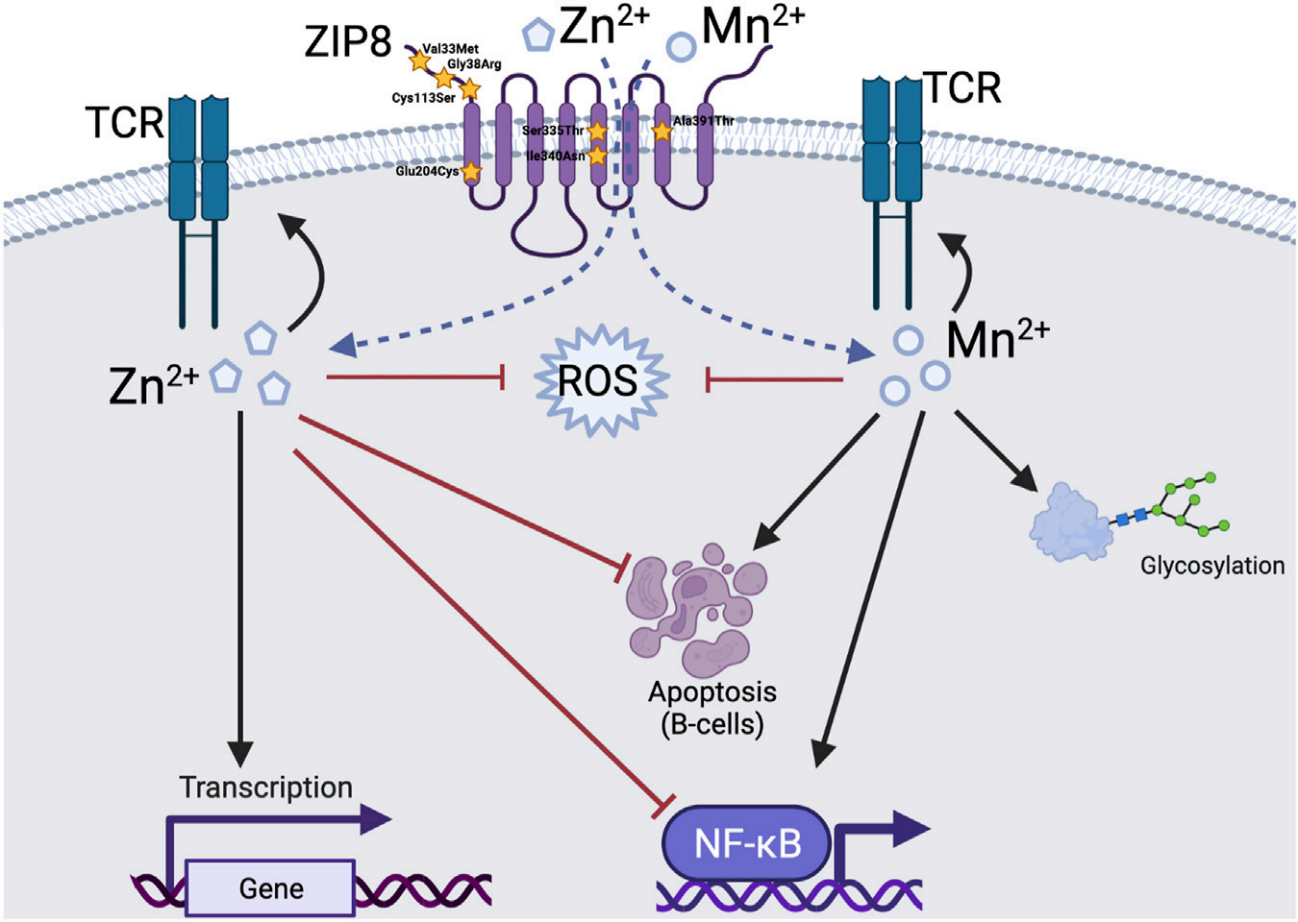
Comparison of the roles of zinc and manganese in immune-mediated host defence against infection. Zn deficiency is common worldwide and physiological Zn concentrations protect against infections by: triggering a variety of transcription factors involved in the immune response, preventing apoptosis of B cells and inhibiting the NF-κB pathway following intracellular transcript *via* ZIP8. In contrast Mn deficiency is rare and it helps protect against infection but different mechanisms that include acting as cofactor for glycosyltransferase enzymes essential for post translational protein glycosylation, enhancing apoptosis of B cells and activating the NF-κB pathway. Whereas each metal has contrasting and distinct functions, they both have antioxidant properties and help facilitate T cell receptor (TCR) signalling. Stars indicate the common polymorphic variants of ZIP8. Red arrows indicate inhibition, black arrows indicate activation.

In addition, in humans that carry defective ZIP8 alleles, can Zn or Mn supplementation at supraphysiologic doses overcome deficits in intracellular metal composition thereby correcting immune function to eradicate infection? This is important to understand given that the biologic footprint of Mn and Zn are in many cases, distinctly different and in some instances, opposite of one another (Figure 1). For example, Zn has been shown by a variety of investigators to inhibit the NF-κB signaling pathway whereas Mn has been shown to augment signaling activity. Likewise, Zn has been shown to inhibit B-cell apoptosis and Mn has been shown to enhance B-cell apoptosis. At the cellular level, we have yet to fully characterize different upstream signals that determine where ZIP8 needs to be at the correct time and what it must do as a function of extracellular or intracellular signals to afford protection to the host. Further, it remains to be determined whether the rate of metal transport and whether ZIP8 can be instructed to distinguish preference toward individual metals in a given context in specific cell types that regulate immune function.

Whether ZIP8 has any role in controlling the dichotomous influence of both metals is not known, let alone other transporters that are capable of mobilizing both metals. Finally, the impact of Mn and Zn transport and cellular function is much more far reaching than individual cells and tissues. Both have influence on microbial communities within the host, not limited to the gut, that determine the composition of and number of microbes as well as the chemical mediators that they produce. More recently it has been shown that alteration of metal content can have profound impact on the host and that in metal deficient states, increase vulnerability of the host to leading pathogens within the gut or other tissues such as lung. Collectively, continued advancements in this area are warranted to better understand the impact of Mn and Zn (in addition to other trace metals) on the host response to dangerous pathogens and by doing so, improve surveillance and therapeutic strategies to prevent or treat infectious diseases.
