# Supplementary material for: Divalent Metal Uptake and the Role of ZIP8 in Host Defense Against Pathogens
Source: Front Cell Dev Biol. 2022 Jun 27;10:924820. doi: 10.3389/fcell.2022.924820 (PMC9273032; doi:10.3389/fcell.2022.924820)
Supplement: Supplementary file 1 [file DataSheet1.PDF]

Single Nucleotide Variant	Minor Allele Frequency	Reference (DOI)
Ala.391.Thr	• 0.05 in American populations<br>• 0.08 in Northern European populations<br>• 0.14-0.25 in Ashkamzi Jewish populations<br>• Monomorphic in African and South Asian populations	10.1172/jci.insight.140978
Gly.38.Arg	• 0.0001255 in European populations	10.1016/j.ajhg.2015.11.003
Ile.340.Asn<br>Val.33.met<br>Ser.335.Thr<br>Gly.204.Cys<br>Cys.113.Ser	• Unknown frequency<br>• Identified using penetrant autosomal recessive models with a rare disease allele frequency of 0.0001	10.1007/s10545-016-0010-6<br>10.1016/j.ajhg.2015.11.002<br>10.1016/j.ajhg.2015.11.003
